# Improved Dividend Estimation from Intraday Quotes

**DOI:** 10.3390/e24010095

**Published:** 2022-01-07

**Authors:** Pontus Söderbäck, Jörgen Blomvall, Martin Singull

**Affiliations:** 1 Department of Management and Engineering, Production Economics, Linköping University, 581 83 Linköping, Sweden; pontus.soderback@liu.se (P.S.); jorgen.blomvall@liu.se (J.B.); 2 Department of Mathematics, Linköping University, 581 83 Linköping, Sweden

**Keywords:** big data adaptation, dividend estimation, options markets, weighted least squares

## Abstract

Liquid financial markets, such as the options market of the S&P 500 index, create vast amounts of data every day, i.e., so-called intraday data. However, this highly granular data is often reduced to single-time when used to estimate financial quantities. This under-utilization of the data may reduce the quality of the estimates. In this paper, we study the impacts on estimation quality when using intraday data to estimate dividends. The methodology is based on earlier linear regression (ordinary least squares) estimates, which have been adapted to intraday data. Further, the method is also generalized in two aspects. First, the dividends are expressed as present values of future dividends rather than dividend yields. Second, to account for heteroscedasticity, the estimation methodology was formulated as a weighted least squares, where the weights are determined from the market data. This method is compared with a traditional method on out-of-sample S&P 500 European options market data. The results show that estimations based on intraday data have, with statistical significance, a higher quality than the corresponding single-times estimates. Additionally, the two generalizations of the methodology are shown to improve the estimation quality further.

## 1. Introduction

This paper presents a method for extracting dividend information from the equity derivatives market using exchange-traded European-typed call and put options. The central methodology in this paper is an extension of the work of Desmettre et al. [1], that is, to formulate a linear regression with a well-known put–call parity. Moreover, we present a novel option position (the sloped asset position), from which it is possible to compute a dividend estimate without specifying an interest rate. Furthermore, throughout the paper, the primary application in mind for the estimates is derivative pricing. This application framing may prima facie seem like an unnecessary limitation, but we argue that the estimates have often-overlooked inherent assumptions that should be aligned with the application. The derivative pricing application follows naturally, whereas other applications require non-trivial adjustments.

One research question is the connection between an asset and its dividends. One of the earliest examples is the asset valuing method: discounted cash flow. The principle idea of that method is that there is a relationship between the price of an asset and its future dividend payments. A related research question is to understand the effect on asset prices of dividend payments. The price of a dividend-paying asset in a frictionless market, ceteris paribus, would drop when a dividend is paid, and the size of the drop would be the size of the dividend payment, see, e.g., Campbell and Beranek [2] and Miller and Modigliani [3]. However, this theory is not supported in empirical studies, and the price generally drops less than the size of the dividend. Campbell and Beranek [2] attribute the differences to tax effects. This idea was elaborated into a formula by Elton and Gruber [4], where the differences between dividend and capital gain taxes were key. Other explanations have been presented, such as transaction costs and behavioral effects. The former was studied by, e.g., Kalay [5] and Boyd and Jagannathan [6], and the latter by Hartzmark and Solomon [7]. Practical imperfections such as a time difference between the ex-dividend date and the payment date can also explain this idea, as was claimed by Wilmott [8]. This paper neither elaborates upon the discounted cash flows method nor provides explanations of imperfect drops in asset prices. Still, these effects must be considered when estimating dividends and evaluating these estimates. We present the implications of the imperfections for estimate interpretation and how to evaluate estimates accordingly.

Dividends are also central in the derivative pricing literature. Dividends have recently started to be seen as an independent asset class according to Filipović and Willems [9], who also provide an overview of this market. This asset class has some interesting properties, but it is not used in this paper. We elaborate on this decision in Section 2.1. Instead, we follow the traditional focus, which has been on modeling the effect on asset price of dividend payments. One of the first to incorporate dividends in derivative pricing was Merton [10], who modeled dividends as continuous adjustments. Another approach is to have discrete adjustments. Discrete adjustments can be applied either as an adjustment of the spot price or an adjustment of the price as time evolves, where the former is sometimes known as an escrowed model (for an overview of this model, see Haug et al. [11], Frishling [12], and Vellekoop and Nieuwenhuis [13]). The use of discrete adjustments is limited since these models have drawbacks. The former contains the possibility of arbitrage opportunities and logical flaws, while the main problem with the latter is its complexity, which often leads to costly methods, see a more elaborated discussion in Haug et al. [11] and Vellekoop and Nieuwenhuis [13]. These problems can be avoided by following Merton [10] and modeling the dividend as a constant continuous yield for each period of maturity, even though that is a poor representation of reality. For example, that method has been applied to models based on stochastic differential equations, such as Carr and Madan [14], Duffie et al. [15], and Carr et al. [16]; implied volatility models such as Gatheral and Jacquier [17]; and local volatility models such as Derman and Kani [18], Derman and Kani [19], and Geng et al. [20].

Regardless of the method, a critical concept is making pricing consistent, which is a critique against the escrowed approach. Even so, estimating dividends, either as a yield or as a present value of future dividends, from market data is not well-studied in the literature. The estimation method that we propose does naturally handle consistent pricing. Furthermore, we study the difference between estimating yield and present value and find that the latter is preferable, regardless of the choice of pricing model. We explain this performance difference in the inherent connection between the dividend yield and the price of the underlying asset.

Although there has been little effort to estimate the dividends for the derivative pricing perspective, it has received more attention in other fields. For example, dividends have long been of interest in studies, such as Fama and French [21], on how dividend yields predict stock returns. Fama and French [21] were not the first to take an intererest in this topic; for an overview of preceding work see their paper and for succeeding papers see Golez [22]. The aims of these papers are of limited relevance in this current study, and relevance is how dividends are estimated. Earlier papers used historical (realized) dividends, but Golez [22] claims that using those could decrease predictability and argued further that inferring dividend yields from the derivatives market is beneficial. Bilson et al. [23] complemented the work of Golez [22] by introducing a novel approach to dividend growth rates implied by market data. Important to note is that Fama and French [21], Golez [22], and Bilson et al. [23] had other aims than to develop a dividend estimation methodology.

Our focus, i.e., on estimation methodology, is not common, but another similar exception is the linear regression (ordinary least squares) methodology presented by Desmettre et al. [1]. In this paper, we generalize the work of Desmettre et al. [1]. The work of Desmettre et al. [1] and our paper can be seen as a parallel to recent work in interest rate estimation by Azzone and Baviera [24] and Blomvall et al. [25]. The estimation methodologies are similar for interest rates and dividends, but the latter contains additional nuances that must be considered. Papers that have estimated dividend quantities from data, such as Golez [22], Bilson et al. [23], and Desmettre et al. [1], have all based their estimates on data from a single time. We expand the data —in the same way as Blomvall et al. [25] does —to use intraday data and find that it provides more stable estimates, i.e., less sensitive to market noise. Moreover, intraday data introduces a coupling to market dynamics that must be considered via a slight reformulation of the regression developed by Desmettre et al. [1]. Additionally, we also present a generalization in the form of weighted least squares formulations.

The estimation methodology is one part of this paper. Furthermore, Desmettre et al. [1] argue that their method and results are limited to markets that meet specific conditions, e.g., the French and German equity markets. This paper presents another interpretation of the quantities, enabling us to evaluate the dividend estimates for more markets, e.g., the US S&P 500 equity market. However, our method is not applicable when used along with equity shares since our methodology relies on relationships between European-typed options. One key of the result evaluations is that the sloped asset position is introduced, acting as an independent method. This position is analogous to the box position used in interest-rate estimation.

The remaining section of this paper is arranged as follows. First, we start with the modeling of dividends, where different estimation methods are also presented. We continue by discussing our data set: the raw data used in the studies and the processing that we performed on the data. In the subsequent section, we present our evaluation methodology, numerical results, and related discussions. Finally, the paper ends with a conclusion and summary of the results found in the paper.

## 2. Dividend Modeling and Estimation

A dividend payment is a way to distribute value from companies to their shareholders. The basic dynamic that we utilize in our methodology is that the asset price drops when the asset pays a dividend. To obtain a forward-looking estimate, we use the derivatives market. To schematically exemplify this, assume that we have a—highly theoretical—situation with two European-styled call options with identical contract specifications, i.e., identical time to maturity, strike price, and underlying asset, but one option has an underlying asset that pays a dividend while the underlying asset of the other option does not. The option with the dividend-paying asset has a lower price than the other since its payoff at expiry is smaller. In this highly theoretical—but unrealistic—setting, we could infer the dividend effect from the price difference between the options. It is possible to achieve a similar inference in a realistic setting by utilizing the derivatives market.

The idea is simple, but the interpretation of the estimated quantity—even in the idealistic setting of the above example—is rather complex. First, in the example above, the difference between the two option prices is not the dividend, since the option owner is not entitled to the dividend in either case. The difference is instead dependent on how the asset price reacts to dividend payments. This insight is—according to us—not sufficiently pronounced in the dividend estimation literature. Nevertheless, it is of significant importance when interpreting estimates.

The difference between drops and dividends has been empirically studied for shares, and different explanations have been proposed. This research question has not been closed, but when we look into options with an index as the underlying, we argue that other additional effects may also be present. The most apparent difference between a single share and an index is that the latter is neither a traded asset nor pays dividends. The value of an index, i.e., the quoted index value, is not a traded price but a computation from the index constituents’ prices according to an index methodology. From this computation, it follows that the index quote should, ideally, experience a drop that reflects the constituent’s equity price drop and weight. There might also be details in the index methodology that further complicate the situation. For example, the S&P 500 index quote is not adjusted for standard cash dividends but extra cash dividends. Hence, in theory, the type of dividend payment is reflected differently in the index quotes. These complications make the estimate interpretation more complex than for a single share.

To summarize, the crucial insight is that the effect seen in the market is not only due to the dividend, but is a result of a mixture of the dividend and its imperfections. Despite the importance of this insight, it has received little attention. Desmettre et al. [1] present a related argumentation, but they limit the imperfections to the tax situation. We instead argue that the estimates should not perfectly reflect the realized dividends but rather a latent quantity. This claim is similar to the claim of Desmettre et al. [1], but the difference is that we do not see tax as the sole imperfection. Nevertheless, throughout this paper, we do not explicitly clarify this point repeatedly and refer to the quantity simply as a dividend to increase readability.

The initial theoretical situation—with two different behaviors, i.e., prices, of the same underlying asset—is impossible to replicate in reality. The key to making the idea usable in reality is creating derivative positions related to the underlying asset. In the following two sections, we first discuss data and different relationships that can be used, and then we discuss how to infer estimates from the relationships.

### 2.1. Market Dividend Relations

The aim of this paper is to create derivative relationships, or positions, of—exchange-traded contracts—from which it is possible to infer the dividend. The relationships that we use should fulfill two properties. First, the data quality of the position should be high, and, second, the position should not require complex modeling of, e.g., the underlying asset, but rather suffice with few assumptions. The former is not a strict definition, but we regard quality as a synonym to liquidity in this paper, i.e., high liquidity is high quality. The reason that we want high-quality data is to ensure that the data quality does not limit the estimates. The limitation of the second property comes from an interpretation of the estimates. The drawback of complex modeling is that the dividend is strongly coupled to the specific model. Estimating the dividend from such a model requires a calibration of the other model parameters. In essence, this coupling makes the dividend an additional model parameter in the calibration process, and, hence, the dividend is affected by the other parameters. This may be a valid method for the calibration of the model, but the dividend is not transferable to other models or applications. Thus, the market contracts we considered in this study were limited by the two properties: high-quality data and non-complex modeling.

The traditional market, when inferring dividend,s has been the equity derivatives market, such as equity futures or equity options. An alternative that may seem attractive is the dividend derivatives market, because of its close connection to dividends, and also because it was used to infer dividend information by van Binsbergen et al. [26]. The market is interesting, but we see three drawbacks of using this market for dividend estimation. First and most important, the underlying of these derivatives is a dividend point index, which is computed from realized dividends. Therefore, the inherent information in the dividend derivatives is linked to realized dividends rather than the effect dividends have on the equity index. This discrepancy makes the dividend derivatives market ill-suited for our estimation since we want to estimate the effect of the asset rather than the dividend. Second, van Binsbergen et al. [26] introduced a model that makes the corresponding estimates less tractable and violates our second property. Additionally, the method used has been questioned by Tunaru [27], who argues that van Binsbergen et al. [26] fail to recognize that dividend derivatives are part of an incomplete market and, thus, that results pbtained using them are invalid. Third, the asset class is not well developed in most markets, and its liquidity is low.

To avoid both illogical approaches and poor liquidity, we use the equity market. In theory, a wide range of derivatives could be used, from plain vanilla to exotic contracts. However, we exclude contracts in the latter category since they require pricing models or have low illiquidity. To conclude, in this study, we considered futures contracts and plain vanilla call and put options to infer dividend information without introducing models and using liquid market data.

The literature for estimating dividends from market data has had two prevailing contract types: futures contracts and plain vanilla European options. The relationships that typically relate to these contracts are the future-basis and the put–call parity. Variations of these positions have been presented, but the common denominator is that they can be constructed almost exclusively and uniquely with exchange-traded contracts. The only component of the relationships that is not directly market observable is the spot interest rates, which match the periods of maturity of the contracts. These unobservable interest rates must be computed from market data. This computation and the corresponding contracts are undesirable because of their increased complexity and reduced tractability.

#### 2.1.1. General Notation

This paper works with two dividend formulations: a yield formulation and a present value formulation. We let δ(t;T) denote the dividend yield estimated at time t for the period [t,T] and $D(t;T_{1},T_{2})$ denote the estimated present value at time *t* of dividends paid in the period $\left[T_{1},T_{2}\right]$. To simplify the notation—when the start of the period coincides with the time of estimation—we also introduce D(t;T)≡D(t;t,T). Another key component in the dividend estimation is the continuously compounded interest rate. We use the formulation used by Blomvall et al. [25] and decompose the interest rate into two terms, one risk-less interest and an additional spread. We denote the risk-less interest rate and the spread at time *t* for the period [t,T] as $r_{o}\left(t;T\right)$ and s(t;T), respectively.

This paper considers options with S&P 500 as their underlying, where the standard S&P 500-option contract is of the European-type. The choice of European-typed options was not made inadvertently. Our method does not hold, in general, for American-typed options. European call and put options are used, but they are always considered in a pair as synthetic forward positions. A synthetic forward position is created from a call–put option-pair, i.e., two options with the same underlying, the same time to maturity, and the same strike price. A long (short) synthetic forward position is equivalent to a long (short) call option position and a short (long) put option position. The name synthetic forward position stems from the payoff, which is similar to a standard forward contract, i.e., linear in the price of the underlying. The payoffs are similar, but there are differences between a standard forward contract and the synthetic forward position. The former is unique for each time of maturity, and, upon entering, the two parties agree on a forward price that marks the contract to the market, i.e., no money is transferred upon entering. The synthetic forward position, on the contrary, is not unique for each time of maturity, and it is possible to specify the strike prices. Thus a money transfer can be necessary to mark the contract to the market.

The quote of the S&P 500 index is computed and presented as a unique value, but the market prices for tradable financial assets are only precise down to a bid–ask spread. Despite this market feature, we formulated all the relationships with a unique price in the remainder of this section. Details are discussed in Section 3, but the unique prices used were mid-prices, i.e., the arithmetic means of the bid and ask prices.

#### 2.1.2. Future-Basis

The future-basis is the relationship between a future and spot price for a futures contract. This position is, in essence, used by Andersen and Brotherton-Ratcliffe [28], and it is also described in various textbooks and practitioner-geared literature, such as Wilmott [8] (p. 1040). Let S(t) denote the spot price at time *t*, and F(t;T) the future price at time *t* with a time of maturity *T*, then the future-basis can be written as

(1) $$\begin{array}{cc} F(t;T) & =S\left(t\right)e^{(r_{o}\left(t;T\right)+s\left(t;T\right)-\delta \left(t;T\right))\left(T-t\right)} \end{array}$$

and

(2) $$\begin{array}{cc} 1-1F(t;T) & =S\left(t\right)e^{(r_{o}\left(t;T\right)+s\left(t;T\right))\left(T-t\right)}-D\left(t;T\right), \end{array}$$

where the former holds for the dividend yield and the latter for a present value of dividends. These relationships can be rewritten as dividend estimates:

(3) $$\begin{array}{cc} \hat{\delta }\left(t;T\right) & =\frac{1}{T-t}\ln\left[\frac{F(t;T)}{S(t)}\right]-\left(r_{o}\left(t;T\right)+s\left(t;T\right)\right) \end{array}$$

and

(4) $$\begin{array}{cc} \hat{D}\left(t;T\right) & =S\left(t\right)e^{\left(r(t;T)+s(t;T)\right)\left(T-t\right)}-F\left(t;T\right). \end{array}$$

One clear advantage of basing dividend estimates on the future-basis is that the estimates are uniquely specified. On the other hand, we see three drawbacks to basing the estimation of the future market. First, the interest rate must be determined, and potential misspecification affects the dividend estimate. Second, the liquidity of the futures contract is only high for short times to maturity, and, hence, estimations corresponding to longer times to maturity are challenging. Third, the uniqueness of the estimate comes with a drawback. To rely on a single contract for an estimate makes it fragile to noise in the futures price. The second and third drawbacks can be resolved using the options market, e.g., via the put–call parity. Moreover, all three drawbacks can be removed entirely with the sloped asset position, but at the cost of the non-unique estimates. It is also possible to mitigate the first and third problem using a suitable estimation method, which is discussed in Section 2.2.1.

#### 2.1.3. Put–Call Parity

The put–call parity is a relationship that relates the price of a European call option, the price of a European put option, and the price of their underlying asset. The put–call parity does not hold for American call and put options since American-typed options can be exercised early, i.e., prior to maturity. This optionality provides the American-typed options a premium that violates the parity. However, Kragt [29] presents a methodology to estimate these premiums simultaneously with the dividend component, which is outside the scope of this paper. To base dividend estimates on the put–call parity is not a novelty. Additional examples are van Binsbergen et al. [30], Hull [31], and Desmettre et al. [1], where the second formulates the parity with a dividend yield and the other two with the present value of the dividends. We let c(t;K,T) and p(t;K,T) denote the European call and put option prices, respectively, at time *t* of options, with strike price *K*, and time of maturity *T*. The put–call parity formulated with a yield and a present value can be written as:

(5) $$\begin{array}{cc} c(t;K,T)-p(t;K,T) & =S\left(t\right)e^{-\delta (t;T)(T-t)}-Ke^{-(r_{o}\left(t;T\right)+s\left(t;T\right))\left(T-t\right)}, \end{array}$$

and

(6) $$\begin{array}{cc} c(t;K,T)-p(t;K,T) & =S\left(t\right)-D\left(t;T\right)-Ke^{-(r_{o}\left(t;T\right)+s\left(t;T\right))\left(T-t\right)}, \end{array}$$

respectively. The left-hand sides of the two relationships can be identified as synthetic forward positions, which we denote as f(t;K,T)≡c(t;K,T)−p(t;K,T). From the parities and fixed *t*, *T*, and *K*, it is possible to find direct formulas of the dividend estimates:

(7) $$\begin{array}{cc} \hat{\delta }\left(t;T\right) & =-\frac{1}{T-t}\ln\left[\frac{c\left(t;K,T\right)\;-\;p\left(t;K,T\right)\;+\;Ke^{-(r_{o}\left(t;T\right)+s\left(t;T\right))\left(T-t\right)}}{S(t)}\right] \end{array}$$

(8) $$\begin{array}{cc} \; & =-\frac{1}{T-t}\ln\left[\frac{f\left(t;K,T\right)+Ke^{-(r_{o}\left(t;T\right)+s\left(t;T\right))\left(T-t\right)}}{S(t)}\right], \end{array}$$

and

(9) $$\begin{array}{cc} \hat{D}\left(t;T\right) & =S\left(t\right)-Ke^{-(r_{o}\left(t;T\right)+s\left(t;T\right))\left(T-t\right)}-c\left(t;K,T\right)+p\left(t;K,T\right) \end{array}$$

(10) $$\begin{array}{cc} \; & =S\left(t\right)-Ke^{-(r_{o}\left(t;T\right)+s\left(t;T\right))\left(T-t\right)}-f\left(t;K,T\right), \end{array}$$

for the yield and present value, respectively. All estimates for a given time of maturity should, in theory, be the same irrespective of the strike prices. This unity is not true in practice, and the estimates differ for different strike prices. These multiple estimates mitigate the fragility of a single contract but at the cost of non-uniqueness. If a single-valued estimate is necessary, we require an aggregation method. Additionally, the options market is more liquid than the futures market for most times of maturity. The exception is short times to maturity, where the futures market is more liquid than the options market. The third drawback of the future-basis (the need for an interest rate) is also present for the put–call parity. One option to remove the need is to utilize a new option position—the sloped asset position.

#### 2.1.4. Sloped Asset Position

Ronn and Ronn [32] presented an option position, the box-position, from which a market-implied interest rate could be estimated without specifying a dividend. The position has been used in the literature, e.g., van Binsbergen et al. [33] and Blomvall et al. [25]. The box-position is constructed by combining two put–call parities or the equivalent of two synthetic forward positions. We build upon the same logic but choose the number of synthetic forward contracts differently. Let $K_{1}\in R^{+}$ and $K_{2}\in R^{+}$, and let the new position consists of one long position in a synthetic forward with the strike price $K_{1}$ and $K_{1}/K_{2}$ short synthetic forward positions with the strike price $K_{2}$. We refer to this position as the sloped asset position, where the name stems from the payoff of the position. From (5) and (6), we can write two relationships (see Appendix A for details):

(11) $$\begin{array}{cc} \frac{f\left(t;K_{1},T\right)K_{2}-f\left(t;K_{2},T\right)K_{1}}{K_{2}-K_{1}} & =S_{t}e^{-\delta (T-t)} \end{array}$$

and

(12) $$\begin{array}{cc} \frac{f\left(t;K_{1},T\right)K_{2}-f\left(t;K_{2},T\right)K_{1}}{K_{2}-K_{1}} & =S_{t}-D\left(t;T\right), \end{array}$$

respectively. We note that the left-hand sides are the same but that the right-hand sides differ, and we introduce the concept of an adjusted spot price, to simplify the notation, thus:

(13) $$S^{*}\left(t;K_{1},K_{2},T\right):=\frac{f\left(t;K_{1},T\right)K_{2}-f\left(t;K_{2},T\right)K_{1}}{K_{2}-K_{1}}.$$

It is possible to reformulate (11) and (12) with the adjusted spot price into:

(14) $$\begin{array}{cc} \hat{\delta }\left(t;T\right) & =-\frac{1}{T-t}\ln\left[\frac{S^{*}\left(t;K_{1},K_{2},T\right)}{S(t)}\right], \end{array}$$

and

(15) $$\begin{array}{cc} \hat{D}\left(t;T\right) & =S\left(t\right)-S^{*}\left(t;K_{1},K_{2},T\right), \end{array}$$

respectively. The advantage of the position is twofold. First, it is less exposed against noise since it—similar to the put–call parity—is not based on a single data point. Second, contrary to the put–call parity and the future-basis, it does not need an interest rate specification. The reduced noise exposure comes with two drawbacks since it is possible to construct many positions. First, similar to the put–call parity, the estimates must be aggregated if a single-value is wanted. Second, the method is unfeasible for some data sets that the other relationships could manage. For example, with a data set consisting of *n* option pairs for a given time of maturity (i.e., *n* synthetic forward contracts), it is possible to construct n(n−1)/2 different sloped asset positions and thus equally as many estimates. This quadratic relationship makes the position computationally unfeasible for data sizes that are feasible for the future-basis and the put–call parity. A solution to this infeasibility problem is to limit the data set, but we have chosen not to limit it, because it is difficult to make such a limitation generally and systematically.

### 2.2. Estimation Methods

The three relationships: the future-basis, the put–call parity, and the sloped asset position could all be used to estimate a dividend quantity, either a yield or a present value, for specific times, *t*, and times of maturity, *T*. The estimation method aims to produce a single estimate for each date, but we have multiple times for every date. Furthermore, the future-basis implies a unique estimate for each time of maturity, while multiple estimates can be inferred from the other two relations. For practical applications, multi-valued estimates do not suffice, and a necessary element in the estimation method is aggregation.

A straightforward approach to produce a single estimate is to limit the data. In doing this, the aim of the method is met, but the drawback is that the technique probably introduces additional noise in the estimates, which comes from the fact that the chosen data points can imply biased estimates. An alternative could be to select data points such that the noise is reduced. The disadvantage of such an alternative is twofold. First, it is challenging to design a method that makes this selection possible. Second, it is a strong assumption that a few data points are representative of the whole market. Therefore, we can adjust the estimates instead of adjusting the (input) data. A technique that would consider all available data points to aggregate the estimates, could, e.g., be a mean or a median computation. The drawbacks of this approach are that it requires that the interest rate is specified exogenously, and that the weights given to specific estimates are arbitrary. For example, in the case of the median, all of the weight is put on a single estimate. To mitigate these drawbacks, we followed the method used by Desmettre et al. [1] and formulated the put–call parity as a linear regression model. Similar formulations have also been used by van Binsbergen et al. [33], Azzone and Baviera [24], and Blomvall et al. [25] for interest rate estimation methodologies.

The regression used by Desmettre et al. [1] is the foundation of our work, but we present three expansions. First, instead of limiting the data used to data from a single time (single-time data), we use data from a whole day (intraday data). Second, we formulate two regressions with different modeling of the dividend: one where the dividend is formulated as a yield and one where it is formulated as a present value. Third, we generalize the regression from an ordinary to a weighted least squares model. It would also be possible to formulate a regression from the future-basis, since we use intraday data. We elaborate slightly in the next section, but we do not see it as an appropriate approach, primarily because of the drawbacks presented in Section 2.1.2.

#### 2.2.1. Linear Regression

We formulated one linear regression model for each time of maturity and each put–call parity formulation, (5) and (6). The first regression model was formulated with a dividend yield, and the second used a present-value formulation. In contrast to Desmettre et al. [1], we used intraday data rather than data from a single time. Further, Desmettre et al. [1] correctly point out that by estimating the dividend with regression, the interest rate is estimated simultaneously, eliminating the need for a separate interest rate estimate. Therefore, it may seem strange to reintroduce the estimation need by formulating the interest rate as a sum of an interest rate and an interest spread, but the reintroduction is necessary due to the fact that we use intraday data. The interest rate for a single time is constant, but it is not, in general, constant across a whole day. Consequently, to formulate the regression with a fixed interest will inevitably involve an approximation. To make a more realistic and suitable formulation, we model the spread as a constant and keep intraday dynamics for the total interest rate. The rationale is the same as that used by Blomvall et al. [25], i.e., that the spread is more stable intraday than the risk-less component.

In the formulation, we let $N^{d}$ denote the number of days we estimated the dividends and let *d* denote the day $d\in \{1,\ldots ,N^{d}\}$. Moreover, we let κ denote a pair of one (intraday) time, *t*, and one strike price, *K*, κ=(t,K). For a day *d* and a time of maturity *T*, we collected pairs in a set $H^{d,T}$ and enumerated the pairs as $1,\ldots ,N^{d,T}$, where $N^{d,T}=\left|H^{d,T}\right|.$ (The operator, $\left|\cdot \right|$, denotes the cardinality of the set.) (The order is unimportant, and the pair with index *i* is thus $\kappa_{i}=\left(t_{i},K_{i}\right)$). We also introduced $\tau^{d,T}$, which is the time to maturity computed at the beginning of the day. To compute the time from the beginning of the day, we followed the interest rate market convention. The put–call parity, Equation (5), can then be written as

(16) $$f_{i}^{T}=S\left(t_{i}\right)e^{-\delta^{d,T}\tau^{d,T}}-K_{i}e^{-r_{o}^{T}\left(t_{i}\right)\tau^{d,T}}e^{-s^{d,T}\tau^{d,T}},\;\forall i=1,\ldots ,N^{d,T},$$

where $f_{i}^{T}\equiv f\left(t_{i};K_{i},T\right)$. We introduced $X_{1,i}^{d,T}:=-K_{i}e^{-r_{o}^{T}\left(t_{i}\right)\tau^{d,T}}$ and $X_{2,i}:=S\left(t_{i}\right)$ to simplify the notation. (Note that $X_{2,i}$ neither depends on the day nor the time of maturity.) We wanted to estimate $e^{-\delta^{d,T}\tau^{d,T}}$ and $e^{-s^{d,T}\tau^{d,T}}$, and denoted the corresponding regression coefficients as $\gamma_{1}^{d,T}$ and $\gamma_{2}^{d,T}$. Thus, it is possible to write the linear regression as

(17) $$f_{i}^{T}=X_{1,i}^{d,T}\gamma_{1}+X_{2,i}\gamma_{2}^{d,T},\;\forall i=1,\ldots ,N^{d,T}.$$

It is possible to write a similar regression, based on (6) by introducing $h_{i}^{T}f_{i}^{T}:=-S\left(t_{i}\right)$ and a regression constant, $\gamma_{0}$,

(18) $$h_{i}^{T}=\gamma_{0}+X_{i,1}^{d,T}\gamma_{1},\;\forall i=1,\ldots ,N^{d,T}.$$

To summarize, we can write the financial quantity estimates from the regression estimates as

(19) $$\begin{array}{cc} {\hat{D}}^{d,T} & =-\gamma_{0}, \end{array}$$

(20) $$\begin{array}{cc} {\hat{\delta }}^{d,T} & =-\frac{1}{\tau^{d,T}}\ln\left[{\hat{\gamma }}_{2}^{d,T}\right], \end{array}$$

(21) $$\begin{array}{cc} {\hat{s}}^{d,T} & =-\frac{1}{\tau^{d,T}}\ln\left[{\hat{\gamma }}_{1}^{d,T}\right], \end{array}$$

where ${\hat{D}}^{d,T}$ is the estimate of the present value for the time of maturity *T*. The interest rate spread estimate, ${\hat{s}}^{d,T}$, can be estimated from the regression models (17) and (18), but the estimates are not generally equal, with the exception of the single-time data.

It is possible to see the differences between using intraday data and single-time data in the regressions. The interest rate, $r_{o}^{T}\left(t_{i}\right)$, is fixed when single-time data is used. This fixed interest rate makes the decomposed interest rate form redundant since the sum of $r_{o}{\left(t_{i}\right)}^{T}+s$ is a constant. Further, the spot price, $S(t_{i})$, is also fixed, making it possible to convert the dividend yield estimate to a present value dividend estimate, and vice versa, without loss or distortion of the estimates. This perfect conversion makes the two different dividend formulations redundant. These redundancies are not present when intraday data is used, since neither $r_{o}\left(t_{i}\right)$ nor $S(t_{i})$ is constant, making the decomposed interest rate necessary and the dual regression formulations interesting.

Finally, from (17) and (18), it is easy to see that the future-basis regressions, based on (1) and (2), would follow. The formulation is made possible by the utilization of intraday data rather than a single-time data. Despite the analogue to the put–call parity, the regression has one shortcoming compared to its put–call parity counterpart. The regression coefficient for the dividend yield-formulated regression is the sum of the dividend yield and spread, $e^{\left(s(t;T)-\delta (t;T)\right)\left(T-t\right)}$. Hence, the future-basis could only be used for present value estimates. This shortcoming and the previously mentioned drawbacks are why we do not consider this regression in this paper.

#### 2.2.2. Linear Regression—Weighted Least Squares

In an ordinary least squares formulation all of the data are considered equally important. This implicit assumption is likely to be incorrect since the quality of data points is likely different. The ordinary least squares formulation does not adjust for this difference in data quality and thus has a drawback. One approach to counteract this behavior is to value some data points more and some less. To formulate this mathematically rigorous method, we followed the idea in Blomvall et al. [25] and use weighted least squares. Considering the models (17) and (18), we can formulate the weighted least squares

(22) $$\begin{array}{cc} \; & \min_{\gamma =(\gamma_{1},\gamma_{2})}\sum_{i=1}^{N^{d,T}}w_{i}^{d,T}{\left(f_{i}-X_{1,i}^{d,T}\gamma_{1}-X_{2,i}^{d,T}\gamma_{2}\right)}^{2}, \end{array}$$

and

(23) $$\begin{array}{cc} \; & \min_{\gamma =(\gamma_{0},\gamma_{1})}\sum_{i=1}^{N^{d,T}}w_{i}^{d,T}{\left(h_{i}^{d,T}-\gamma_{0}-X_{i,1}^{d,T}\gamma_{1}\right)}^{2}, \end{array}$$

where $w_{1}^{d,T},\ldots ,w_{N^{d,T}}^{d,T}$ are non-negative weights. Note that if each weight is chosen as a positive constant, i.e., $0<w=w_{1}^{d,T}=\ldots =w_{N^{d,T}}^{d,T}$, we obtain the ordinary least squares estimator, only if w=1, the same sum of square errors, is the same. The crux with these formulations is to determine weights. The key idea of the weights is to choose them such that the resulting estimator has good properties. An essential property for the ordinary least squares estimator is that if the residuals are independent and homoscedastic (same finite variance), the estimator is the blue (best linear unbiased estimator). The residuals from the regressions (17) and (18) likely do not fulfill the homoscedasticity, and an ordinary least squares estimator is not the blue. One reason is that the liquidity of the data varies between strike prices, where illiquidity typically leads to higher variance.

The heteroscedasticity can be counteracted, and it is possible to achieve the blue with a specific weighting scheme. According to Aitken [34] (the result can also be found in textbooks such as Zwanzig and Liero [35]), if the weights are chosen to be inversely proportional to the variances, the estimator receives the blue property. In addition to the statistical properties, Blomvall et al. [25] pointed out that weights chosen inversely to the residuals also have an economic rationale. The residuals can be interpreted as a measure of the repricing capabilities of the linear models, where smaller residuals indicate accurate repricing. Nevertheless, the appealing theoretical property has a practical drawback since the variances are unknown and need to be estimated. Estimating the variance for residuals is non-trivial, since we only have a single residual if we fix the time, strike price, and time of maturity, i.e., we do not have repeated estimates of a quantity. To mitigate this problem, we make the same assumption as Blomvall et al. [25] that the variance is constant intraday, i.e., for a fixed strike price and time of maturity. Hence, the variances can be estimated from different intraday times. The weights are computed with the same four-step processes used by Blomvall et al. [25].

First, an ordinary least squares estimate is computed, and the (raw) residuals are determined, which we, for each strike price and time of maturity, denote as $e_{i}$, $\forall i=1,\ldots ,N^{d,T}$. Second, the residuals, $e_{i}$, are grouped into (index) groups according to their strike prices, $R_{K}^{d,T}=\left\{i|i\in \left\{1,\ldots ,N^{d,T}\right\}\;\mathrm{and}\;K_{i}^{d,T}=K\right\}$. Third, a variance is estimated for each group, where:

(24) $$\begin{array}{cc} \mu_{K}^{d,T} & =\frac{1}{\left|R_{K}^{d,T}\right|}\sum_{i\in R_{K}^{d,T}}e_{i}, \end{array}$$

(25) $$\begin{array}{cc} \nu_{K}^{d,T} & =\frac{1}{\left|R_{K}^{d,T}\right|-1}\sum_{i\in R_{K}^{d,T}}{\left(e_{i}-\mu_{K}^{d,T}\right)}^{2}, \end{array}$$

denote the estimated mean and variance, respectively, for the group associated with the date, *d*, the time of maturity, *T*, and the strike price, *K*. Finally, the weights in (22) and (23) can be determined from the auxiliary weights ${\tilde{w}}_{K}^{d,T}=1/\nu_{K}^{d,T}$, as

(26) $$w_{i}^{d,T}={\tilde{w}}_{K_{i}^{d,T}}^{d,T},\;\forall i=1,\ldots ,N^{d,T}.$$

## 3. Data

The data set used in this paper is the same data set used by Blomvall et al. [25]. All the data have been collected from the data provider Thomson Reuters Refinitiv Eikon, and the data set consists of three types of intraday data. First, quotes of the S&P 500-index. Second, bid and ask quotes of European call and put options with the S&P 500-index as their underlying. Third, payer and receiver quotes of fix rates of USD denoted by overnight index swaps contracts with the federal funds rate as the reference rate.

The tick data is collected for all dates in the period from 1 March 2020 to 31 January 2021 between 9 a.m.–4 p.m. The European options are all the available monthly options for the given dates, i.e., all options expiring on the third Friday of each month. The USD overnight index swaps fix rates have a maturity between 1 and 10 years. The data set consists of 6 million S&P 500 index quotes, 110 million bid and ask quotes of the USD overnight index swaps, and 54 billion option prices.

Although granular, the data set must be processed to be useful in the paper. The collected data has four inherent problems. First, we collected tick data, but it is difficult to use because of its irregularities. The data is transformed to a more usable form where the level of granularity is preserved. Second, the quotes of the fix rates of overnight index swaps are not directly usable since (17) and (18) require continuous spot rates, and thus a transformation is needed. Third, in Section 2, all regressions were formulated with a unique price, but in the data set, the prices are only precise down to a bid–ask spread. Earlier, we mentioned that the price used is the mid-price, and below, we discuss this issue. Fourth, we discuss how to identify and remove unrealistic data points.

### 3.1. Synthetic Forward and Sloped Asset Positions

In Section 2, we used synthetic forward and sloped asset positions with unique prices. Neither of these positions is traded in the market, rather only the options are. Hence, neither synthetic forward nor sloped asset positions have quoted bid or ask prices. To circumnavigate the missing prices of these positions, we first computed their bid and ask prices. The mid-prices were then computed from these bid and ask prices. The bid and ask prices were created by artificially replicating the market prices of entering such positions.

Let *c*, *p*, and *f*, respectively, denote the price of a call option, put option, and synthetic forward; and let *a* and *b* denote the ask and bid price, respectively. The payments of the bid and ask positions can be summarized as $f_{a}=c_{a}-p_{b}$ and $f_{b}=c_{b}-p_{a}$. We compute the mid-price of the synthetic forward as

(27) $$f_{m}=\frac{f_{a}+f_{b}}{2}=\frac{\left(c_{a}-p_{b}\right)+\left(c_{b}-p_{a}\right)}{2}=c_{m}-p_{m}.$$

It is thus necessary to have four prices—bid and ask prices for both the call and put options—to compute the mid-price of the synthetic forward position. Hence, if one or more quotes are missing, the mid-quote is not computable and thus not used in the regression.

We likewise compute mid-prices of the sloped asset position by first computing the bid and ask prices; the argument is analogous to the synthetic forward position. We compute the price of entering the position in two directions, and the mid-price is the average. Let $\phi_{i,j}$ denote the sloped asset position, which is going long in a synthetic forward with strike price $K_{i}$, and short in $K_{i}/K_{j}$ synthetic forwards with strike price $K_{j}$. The cost of entering such a position is the ask price, $f_{i}^{a}$ reduced by the bid price, $f_{j}^{b}$ for each of the $K_{i}/K_{j}$ contracts. The ask price of this contract can thus be written as $\phi_{i,j}^{a}=f_{i}^{a}-K_{i}/K_{j}f_{j}^{b}$. Alternatively, we receive $f_{i}^{b}$ and must pay $f_{j}^{a}$ for each of the $K_{i}/K_{j}$ contracts, and the bid price of the slope position is thus calculated $\phi_{i,j}^{b}=f_{i}^{b}-K_{i}/K_{j}f_{j}^{a}$. The mid-price of the slope position is

(28) $$\begin{array}{cc} \phi_{i,j}^{m} & =\frac{\phi_{i,j}^{a}+\phi_{i,j}^{b}}{2}=\frac{\left(f_{i}^{a}-K_{i}/K_{j}f_{j}^{b}\right)+\left(f_{i}^{b}-K_{i}/K_{j}f_{j}^{a}\right)}{2} \end{array}$$

(29) $$\begin{array}{cc} \; & =\frac{\left(f_{i}^{a}+f_{i}^{b}\right)-K_{i}/K_{j}\left(f_{j}^{b}+f_{j}^{a}\right)}{2}=f_{i}^{m}-K_{i}/K_{j}f_{j}^{m}. \end{array}$$

We see that the data of synthetic forward contracts is sufficient to express both the put–call parity and the sloped asset position.

### 3.2. Transformation and Cleaning of Option Tick Data

Our data management has two aims. First, to make the data appropriate for the (estimation) method, and second, to clean the data from the artifacts. While the former is a necessity, the second can lead to the validity of the method being questioned; hence, the data cleaning is moderate. In this paper, we address two features of this data set: a sudden and temporary downward spike in bid quotes and a lack of bid quotes for out-of-the-money options. We classify the former as a data artifact that normal market dynamics cannot explain, while the second has a natural explanation.

**Figure 1:**
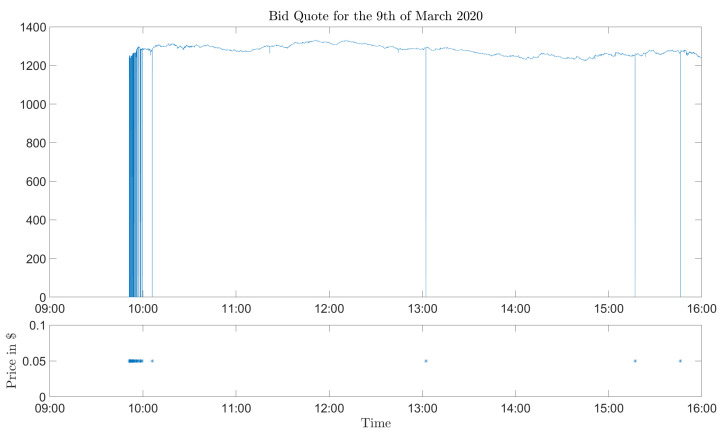
The two panels illustrate the bid quotes of a call option on the 9 March 2020. The strike price of the option is $1500 (in-the-money), and its expiration is the 20 March 2020. The two panels illustrate the same data, but the lower panel focuses on smaller values and thus has a smaller y-axis than the upper panel.

The first problem (downward spikes) is illustrated in Figure 1. We deem the drops of approximately $1200 to be non-realistic and deem further that those spikes have been created in the data collection. We cannot explain why only the bid quotes are affected by this effect. The problem of the downward spikes is easily solved by removing them from the data set. The crux is to determine which of the bid quotes are artifacts and which that are not. In Figure 1, the artifact is evident, but there could be other cases where the spikes are not as obvious. A rough description is that the drops are more pronounced for (deep) in-the-money options than out-of-the-money options, since the former options naturally have higher prices. However, the silver lining is that the effect of the data is less severe, since out-of-the-money options have a lower price; thus, the drops cannot be as big. Therefore, we limited the data cleaning to in-the-money options, since they are more affected and easier to find than out-of-the-money options. The data cleaning procedure that we used was to discard all call (put) options with strike prices greater (less) than the spot price as well as all quotes smaller than $1.

The second problem (missing bid quotes) is not as trivial or obvious as the spikes. Many (deep) out-of-the-money options in the data set lack bid quotes but have corresponding ask quotes. We attribute this data property to the tick size of the market, i.e., the minimum amount that a quote can be changed. This amount may be greater than the fair price of some options, and any (positive) bid quote would thus be overpriced. If the only possible price is an overprice, the only sensible action is not to quote. The ask prices do not suffer from the same dynamics, since it is natural to ask for a higher price than a fair price. The drawback of the estimation method with missing bid quotes is that it decreases the data set significantly. As noted above, a single synthetic forward price mid-price requires both a bid and ask quote for one call and one put option. To reduce the data waste, we recreated the bid quotes.

The strict natural lower limit of plain vanilla option quotes is zero. A price of zero means that someone, essentially, gives away a contract for free with only positive (including zero) payoffs, which is an arbitrage opportunity and, thus, a non-realistic scenario. However, the bid and ask prices are not used individually, but rather only in pairs, to compute mid-prices. Therefore, we argue that when the fair option price is within one tick from zero, it is a valid approximation to set the bid price to zero. A zero bid price is too low, the subsequent mid-price is too low, and a bias is introduced in the mid-prices. In order not to introduce biases, we only replaced missing bid quotes for some call and put options, which are essentially options with small prices. Let *S* denote the intraday median spot price, *K* the strike price, and let $a^{d,T}:=a\sigma \sqrt{\tau^{d,T}}$ where a>0 and σ>0. We replaced missing bid quotes for call and put options if $S(1+a^{d,T})<K$ and $S(1-a^{d,T})>K$, respectively. (The economic interpretation is that bid quotes are only replaced for options with a strike price at least *a* standard deviations, σ, from the current spot price of the underlying asset, i.e., deep out-of-money options.)

Data cleaning is the first step in the data transformation process, where the second is to process the data into a better-suited format. The collected option data is tick data of values and timestamps, which has a precision of one second. One alternative would be to transform the tick data set into a set where the data points are spaced with a fixed time unit, e.g., a second. In essence, the idea is to use the most recent tick quoted in the market for every time unit, using the most recent tick in the case of tick data to second data. The assumption is that, as long as new ticks have not reached the market, i.e., no new information has reached the market, the old ticks are still valid. There are two practical benefits of such an approach. First, it is easy to work with such data. Second, the data utilization is high. Furthermore, if no new information has reached the market, that implies that the market’s dividend and interest-rate beliefs are unchanged. (The converse is not true, changed prices are not synonyms for changes in the markets in terms of the dividend or interest rates beliefs, but it can signify a myriad of factors).

The drawback of such an approach is the risk of amplifying noise. All market information carries some noise, and repeating individual data points would assign higher confidence or weight to arbitrary points and consequently amplify noise in these points. In order not to indirectly assign higher weights to certain points and instead to keep the data utilization high, we are only interested in times where at least one quote of at least one option has changed from the previous time. In this method, the prices of the options should correspond to the index quote for the same times. The transformation that we propose is a two-step process. First, the tick data set is transformed into a set with a specific frequency, i.e., the time between data points, e.g., 1 second, which is the frequency used in this paper. Second, this data set is transformed into the final data set, where only the data points that have changed are kept. Small schematic examples of mock tick data, fixed time unit data, and the final data are presented in Table 1, Table 2 and Table 3, respectively. Note, before the first tick of the day, the quote is written as not available (n/a). The value from a tick prevails until a new tick comes or the day ends (4 pm). The transformation is performed for all options and all fixed rates. The second transformation is from the one-second data to a data set that only contains seconds that coincide with ticks. Table 3 presents a continuation of the example in Table 2. Note that this transformation is not a reversal of the first transformation. The first transformation was made for individual options’ bid and ask quotes, and the second considers all the options’ bid and ask quotes (for a given day and time of maturity) simultaneously.

### 3.3. Overnight Index Swap Implied Spot-Rates

The regression formulations (17) and (18) require continuous spot interest rates. In this paper, we follow the arguments in Blomvall et al. [25] and base these rates on OIS contracts. A specific interest rate is not critical since we estimate a spread over this rate, and most rates are stable intraday. From that point of view, we could have used interest rates from a data provider, such as Thomson Reuters Eikon Refinitiv.

However, the interest rate data must match the frequency of the option data, and thus we must compute them. We use the technique proposed by Blomvall [36], which produces a complete forward-term structure of daily forward interest rates. In this paper, only the spot rates that correspond to the options times of maturity are of interest, and these rates are computed from the forward rates.

## 4. Results and Discussion

This results and discussion section consists of four parts. First, we present the characteristics of the estimates in plots, which are the foundation of the next part in the section. In the second part, in-sample results are presented, that is, results where the data set has not been divided into training and test sets. The in-sample results answer some questions, but the validity of the results can be partly questioned since the results could be the effect of over-fitting. The third part presents the methodology for performing out-of-sample testing, i.e., the data partitioning and evaluation methods. The results include both some basic statistics and a statistical Diebold–Mariano test. Throughout the section, we discuss and highlight results when presented, but one question spans multiple parts—the difference in estimating yield and present value, and hence, it is discussed in the fourth, and final, part of this section.

In addition to the question of the difference between yield and present value, two additional questions are discussed in this section. First, various regressions for dividend estimation have been presented, which can be grouped according to two properties: the weighting scheme and the type of data. The regressions have been formulated generally to handle intraday data, which is similar to the approach used by Blomvall et al. [25]. Contrariwise, in Desmettre et al. [1] and other methodologically similar approaches for interest rate estimation, single-time data is used, see Blomvall et al. [25] for an overview of the latter. From these regressions we make two comparisons. First, we compare the single-time and the intraday dataset. Second, we also study the differences between the weighted least squares and the ordinary least squares models.

### 4.1. Characteristics of Estimates

**Figure 2:**
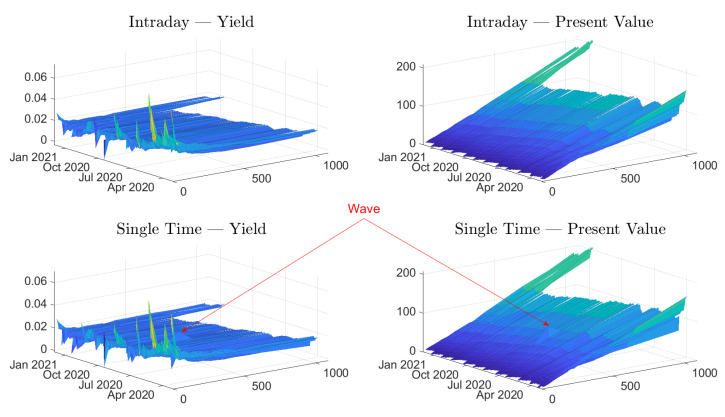
The figure shows the surface plots of the ordinary least squares dividend estimates, which can be grouped by two properties. First, the two upper and two lower panels are computed with intraday and single-time data, respectively. Second, the left and right panels are computed as dividend yields and the present value of dividends, respectively. The z-axes of all four panels indicate the estimated values. The x- and y-axes corresponds to the date and time to maturity (measured in days), respectively, to which the estimates correspond.

**Figure 3:**
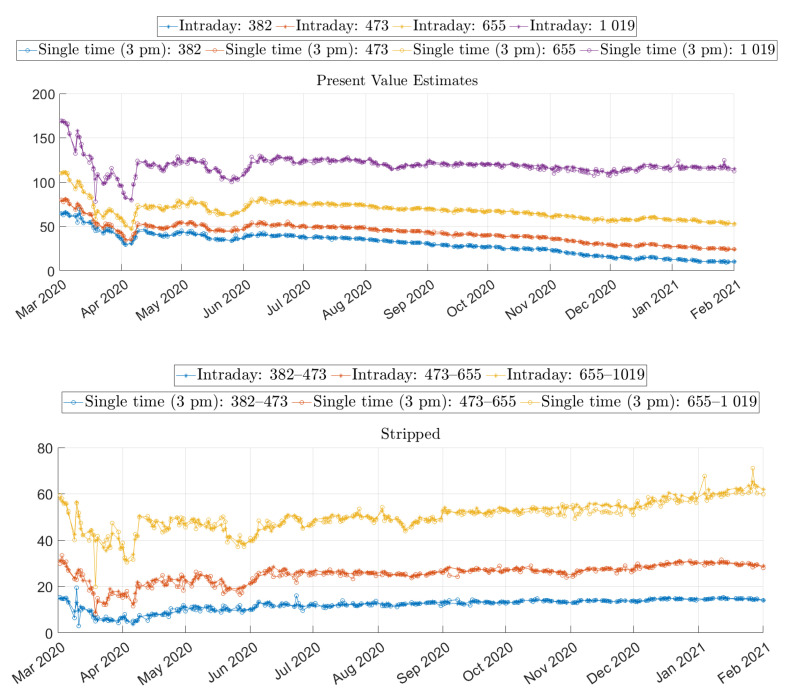
The figure consists of two panels, where the upper and lower panels illustrate the present value dividend estimates that are market-matched and stripped, respectively. The plots illustrate a series of dividend estimates with fixed times of maturity, where the x-axis is the date. The series in each panel consists of either estimates determined by intraday data or by single-time data recorded at 3 p.m. The legend of the upper panels indicates the type of data, and the number is the number of days to maturity at the first date. The legend of the lower panel contain a period, which is the number of days to maturity for the two contracts that have created the stripped dividend.

**Figure 4:**
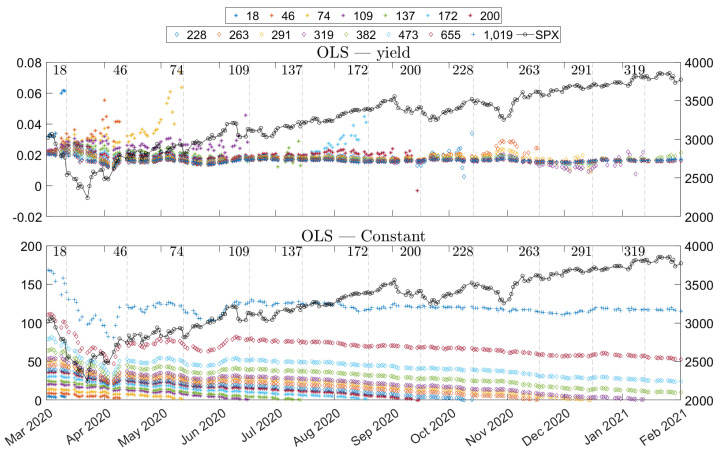
This figure consists of two panels which share the same labels. The upper panel illustrates dividend yield estimates, and the lower panel illustrates present value dividend estimates. The data illustrated in these two panels are part of the data in Figure 2. However, in these two panels, we plot dividend estimates that have a constant time of maturity (one of the dimensions of the surf plots has been removed). The times of maturity that are illustrated are those that were present in the market as of 2 March 2020 (i.e., the first date in our data set). The legends show the time to maturity (measured in days), corresponding to the times of maturity at the first date.

The characterization of the estimates is divided into two parts. First, we have three illustrations of the estimates, both for intraday data and single-time data. The data set is not partitioned in this section, but rather all the data for each time has been used. Second, we start by presenting some surf plots in Figure 2. The surf plots provide an overview, but it is difficult to see any small differences. The line plots in Figure 3 and Figure 4 complement the surf plots.

The overall illustration in the surf plots and the line plots is that the present value estimates have a downward sloping trend as time progresses. These trends, for the longer maturities, are supported by the mean values in Table 4, which indicates that the mean daily changes are negative. These slopes are expected since the present value—for a specific time of maturity—naturally decreases when ex-dividend dates are passed. On the other hand, the yield estimates do not form a slope. Instead, the main effect is that they converge for longer times of maturity. This convergence can be interpreted as expected dividends, in dollars, being stable over the years.

We can observe that both the yield and present value approaches are stable as time evolves, but the estimates vary substantially for different maturities. We can also see an additional effect: estimates drift off shortly before the expiration date. The problem effect is easily observable for the yields in Figure 2 and Figure 4. The effect is also observable for the present value data, but the scale of the plot masks the effect. In most cases, the drift is positive, but we can observe some negative estimates. A negative yield or a negative present value can be interpreted as a cash flow that lifts (negative drop) the price. It is an improbable market dynamic, and, since we experience these negative estimates adjacent to other spurious estimates, we argue that these are not to be taken at face value. Instead, the estimates in these regions should rather be seen as indications of artifacts of the estimation method. A similar effect was reported by Blomvall et al. [25] for interest rate spread estimates, and we follow their argument and explain this effect with low option data quality. Finally, we can see that these spurious values are more pronounced for the single-time data than for the intraday data. We only consider option pairs with a time to maturity exceeding five days to reduce the impact on the results of these spurious values.

The above observation illustrates potential data problems, and provides some insights into how the market reacted during the period. The core idea of the paper is not to understand and study the market dynamics. Nevertheless, the illustrations indicate shifts in the market, which are too significant to leave without comment. The comments are not detailed but instead focus on the holistic picture. In Figure 4, we can see that around April 2020, the estimates behave differently than for the other period, which is a period when the global pandemic started to affect the markets. It is possible to see that the estimates of present value squeezed together, i.e., the difference in estimates of longer and shorter times of maturity reduced. The yield estimates were also affected, but rather with in the opposite direction. The difference between the long and short times of maturity increased. We can also observe that the S&P 500 index quote also experienced a downturn. The effects on the estimates can prima facie seem contrary, but both behaviors have the same underlying reason. During this period, many companies cut, either partly or entirely, their future dividends but kept dividends that were closer in time (e.g., announced dividends), and the market anticipated further cuts for future dividends. For the present value dividend estimates, the effect was direct. Present value dividend estimates corresponding to longer maturities were reduced more than the corresponding short times to maturity. This phenomenon is natural, since both the realized and anticipated dividend cuts were more pronounced for longer times to maturity. A similar effect would have been seen in the yields if the S&P 500 quote had been constant, but the downturn of the S&P 500 offset the effect of lower yields, especially for short times of maturity, and resulted in higher yield estimates for shorter times to maturity. We can see that the estimates have captured these market dynamics and could potentially be a good measure of how the market predicted large dividend cuts.

The interconnection between the yield and the quote of the underlying asset is interesting in two regards. First, it gives rise to counter-intuitive behavior. Second, and more important, from the view of assumptions, if a yield is constant intraday, this would imply highly fluctuating present value dividend estimates during the day. Such behavior seems unlikely from market participants. This view is complemented by Vellekoop and Nieuwenhuis [13], who claim that market makers prefer to specify fixed cash amounts rather than yields. We take this as an indication that the constant dividend yield formulation has inherent problems. In the upcoming sections, we present results that support the fact that yield estimates perform worse than their present value counterparts, and that these differences in performance can be related to the variability of the underlying asset.

### 4.2. In-Sample

We have two types of in-sample results. First, we elaborate on whether the yield or the present value should be used. (This question is also discussed in the next section, where an out-of-sample analysis is performed). Second, we examine the difference between the intraday and single-time data.

An interpretation of the linear models is that it is a pricing method for synthetic forward positions, and an obvious performance measure between the models is to compare the residuals. The residuals are informative but difficult to compare. Therefore, we compare the mean squared errors rather than the residuals themselves in the analyses.

#### 4.2.1. Yield and Present Value Comparison

We consider two different regression models, (17) and (18), where the former is formulated with a dividend yield and the latter with the present values of dividends. The results of the regressions are shown in Table 5, and we can observe that the present value dividend formulation (18) outperforms the yield formulation (17), i.e., the former has a lower mean squared error than the latter.

In Table 5, only the ordinary least squares results are presented, not the corresponding weighted least squares results. The reason is that the ordinary least squares estimator by construction produces a lower mean squared error than the weighted least squares estimator, cf. (22) and (23). Therefore, to have a meaningful comparison, we will compare the ordinary and weighted least squares estimators out-of-sample in the next section.

It is possible to see a difference in predictability between the yield and present value formulation, but it is not easy to relate the two quantities and determine the magnitude of the difference. The yield is transformed into a present value to enable a comparison between the estimates. We represent the yield implied present value with $D_{y}$. The key in the transformation is that both estimates can be interpreted as a spot price adjustments. The yield and present value adjusted spot prices can be written as $S\left(t\right)e^{-\delta (t;T)(T-t)}$ and S(t)−D(t;T), respectively. By equating these two adjusted spot prices, we write the yield-implied present value as

(30) $$\begin{array}{cc} D_{y}\left(t;T\right) & =S\left(t\right)\left(1-e^{-\delta (t;T)(T-t)}\right), \end{array}$$

which can be rewritten into conversions between yield and present value. We can see the results of this conversion in Table 6. The differences between the yield implied and the estimated present value are not big, but there are differences. The statistics in the table do not present any clear differences between the two estimates. The summarized picture shows that the differences are close and symmetric around zero, since the mean values are close to zero with a low standard deviation, while the skewness and kurtosis indicate that there are extreme points. The skewness shows that the implied present value dividend is higher than the present value dividend in eight of eleven ranges and in total. Furthermore, the high kurtosis shows differences notably more extreme than a couple of standard deviations from the mean. The only visible trends in the data are that the standard deviations and the absolute differences seem to increase with longer times to maturity. These results are thus inconclusive as to whether there is a difference or if the estimations only are noisier for longer times to maturity. We continue this discussion around the out-of-sample tests.

#### 4.2.2. Intraday and Single-Time Data

Blomvall et al. [25] concluded that intraday data produce more stable and higher quality estimates than data recorded from a single time. Therefore, we undertook a similar analysis and performed linear regressions where only data points recorded at 3 p.m. were used. First, Table 5 shows mean squared errors for both the intraday and the single-time data set, but the mean squared errors are not directly comparable since the errors are computed from different data sets. Consequently, we do not make any such comparisons in-sample but postpone them to the out-of-sample analysis.

It is possible to consider the surf plots in Figure 2 again. The differences between the intraday and single-time data seem small, but it is possible to observe a wave for both estimate types, which indicates that some estimates differ from adjacent estimates. These estimate differences are more visible in the upper panel of Figure 3 than in Figure 2. We can see that around the period of March–May of 2020, the single-time estimates seems to be more volatile than the intraday estimates. Further, later in the studied period, there are occasional single estimates that are considerably different from their adjacent estimates.

We can study the estimates that correspond to the times of maturity of the options market, which we refer to as market-matched dividend estimates. We want two properties when computing the statistics of the estimates: to estimate the same quantity every day and to have large sample sizes, i.e., long times series of estimates. The latter property is achieved by limiting the data set, such that only the times of maturity that are present in the market for the whole period of study are included. The first property is impossible to achieve completely since the market changes with time. In a period, [t,T], the value of the dividends changes because the ex-dividend dates are passed as *t* evolves. Additionally, the estimated quantity may also change since the beliefs of future dividends change. By studying the present values of the dividends estimates between two maturities that have not passed in the period, the impact of passed ex-dividends dates is removed. We refer to these differences as stripped dividend estimates. We use the notation introduced in Section 2.1.1, where D(t;τ,T) is the present value of dividends within the ex-dividend date in the period [τ,T]. We can then measure some statistics of these stripped dividends, an analysis that is similar to the analysis conducted by Desmettre et al. [1].

The market-matched and stripped dividend estimates are similar, but they have some differences in their interpretations. Statistics of the market-matched estimates can be seen in Table 4, and statistics for the stripped dividends estimates are presented in Table 7. Further, the market-matched and stripped dividends are presented in the lower panel of Figure 3. The stripped dividends estimates do not have the downward slopes that the market-matched dividend estimates have. The line plots of Figure 3 are flat, and the means of Table 7 are approximately zero. The reason for the slope is that the ex-dividend dates are passed for the market-matched dividend estimates, but since the stripped dividends are further in the future, no ex-dividend dates have passed.

The means are similar for both data sets, but the single-time data estimates have higher volatility values than the intraday data estimates. Furthermore, the standard deviation (volatility) values are similar between the market-matched and stripped dividends, which is surprising. The stripped dividends are estimates of fewer dividends than the market-matched dividends, and additionally, those dividends are shared with the market-matched estimates. Therefore, a natural assumption is that the dispersion of the former would be smaller. A possible explanation is that the future dividends are uncertain. Another explanation is that there is noise in the estimates, which may be because options with longer times to maturity are less liquid than options with shorter times to maturity.

Furthermore, the auto-correlation of the daily differences holds interesting information. We can see in Figure 3 that there are some upward spikes for individual days, i.e., it goes up one day and then comes back to a similar level the following day. This pattern is a clear sign of the noise in the estimates. We can contrast the single-time data plots with the plots of the intraday data, which lack clear spikes. We measured the auto-correlation to see how much the estimates were affected and presented the results in Table 4 and Table 7. We noted that the market-matched dividend estimates had a lower auto-correlation since these estimates had a downward trend. This downward trend reduced the information in the auto-correlation, and, thus, the auto-correlation values of the stripped dividends are better indicators of the noise for each method. We can see in Table 7 that the auto-correlation is negative for both the intraday and the single-time data, but the auto-correlations are smaller (more negative) for the latter. The negative sign indicates that both types of estimates are affected by noise, and further, the differences between the auto-correlations indicate that intraday estimates contain less noise than the single-time estimates. Further, it is impossible to make statements concerning the noise level in the market match contra the stripped dividend estimates since the market-matched dividends have a natural downward slope, which thus increases the auto-correlation of the daily differences.

### 4.3. Out-of-Sample

The in-sample results indicate that the present value of the dividends performs better than the yield estimates. However, these results can be questioned, since the performance may be a result of over-fitting. In this section, we perform an out-of-sample analysis. The analysis is a two-step approach. First, we discuss how to partition the data into two sets: the training and test sets. The former was used for estimating, while the latter was used for evaluating the estimates. Second, we present the evaluation method.

#### 4.3.1. Partitioning the Data Set

In order to make an out-of-sample analysis, the data set needed be divided into two parts. The data consisted of all (business) dates from 1 March 2020 to 1 February 2021. Each date had some times of maturity, and linear regressions were performed for each time of maturity. The partitions into in- and out-of-sample sets were performed on each such unit, since there was neither data sharing between the dates nor the times of maturity. The data set used for estimation consisted of three data types: the spot price of the underlying (i.e., quotes of the S&P 500 index), spot interest rates, and synthetic forward mid-prices. The regressions use both different times and different strike prices. We argued in Section 3 that information reaches the market over time and that the times are important. For each (intraday) time, a single and unique S&P 500 quote and a single unique spot rate exist. This uniqueness creates the need for these points to be used both in- and out-of-sample. On the other hand, the synthetic forward prices can be partitioned into two sets.

The partition is performed with two principles. First, we want a wide range of strike prices in-sample since they are important for making good estimates. Second, we want to have a greater portion in-sample than out-of-sample. The set of synthetic forward positions is divided into an in- and out-of-sample set according to two criteria. The first criterion is that a synthetic forward is included if its strike price is below a lower limit, $\ell \in R^{+}$, or above an upper limit, $u\in R^{+}$. The second criterion is that of the synthetic forwards not included in-sample by the first criterion, every *k*:th is placed in the out-of-sample set, while the remaining are placed in-sample, where $k\in N^{+}$, i.e., a strictly positive integer. It would be improper to make the first inclusion criterion static, since the index value changes during the studied period, and thus the limits of in- and out-of-money change. Therefore, rather than assigning static values to *ℓ* and *u*, we assign values relative to the index value for each day. The index value was not constant intraday, and we computed the index daily reference value as the median of all intraday index quotes and denoted it with $\hat{S}$, and we defined $\ell =\ell^{\prime }\hat{S}$ and $u=u^{\prime }\hat{S}$, where $\ell^{\prime }\in R^{+}$ and $u^{\prime }\in R^{+}$.

The in-sample and out-of-sample data are from the same data set, but their roles are not equal. The in-sample should, in essence, be the data used for estimation. The out-of-sample, on the other hand, was used as a reference, and we could have been more selective when forming this set, and, e.g., used additional filters. One rough measure of the quality of prices is the size of the bid–ask spread, where a wide spread indicates a less reliable price and a narrow spread a more reliable price. The idea is to remove options with too wide spreads, an idea which was used by Blomvall et al. [25] and Azzone and Baviera [24]. The crux is to characterize a typical and reasonable spread. One natural dynamic to keep in mind is that options with higher prices have wider spreads than options with lower prices, if the spreads are measured in an absolute dollar amount. This relationship means that in-sample options have wider spreads than out-of-sample options, and options with longer maturity times have larger spreads than options close to expiry. However, this dynamic is not a big problem in practice. The latter is not a problem, since each time of maturity is managed independently. The former is slightly more challenging, but since the out-of-sample is a subset in which deep in- and out-of-sample options have been excluded, the potential impact is limited. Further, the spreads can also vary between days, and thus they are not suitable to use as a fixed cutoff value. Instead, a reference is computed for each date to account for this variability.

The additional filter handles call and put options separately and are applied for each date and time to maturity. Let, $N^{d,T}$ denote the number of option pairs for date *d*, with time of maturity *T*, and let $\Delta c_{i}=c_{a}^{i}-c_{b}^{i}$ and $\Delta p_{i}=p_{a}^{i}-p_{b}^{i}$ denote the spread of the *i*th call option and put option, respectively. The scaled median of the spreads is computed as $m_{c}=\left(1+b_{c}\right)\underset{i\in \{1,\ldots ,N^{d,T}\}}{\mathrm{median}}\Delta c_{i}$ and $m_{p}=\left(1+b_{p}\right)\underset{i\in \{1,\ldots ,N^{d,T}\}}{\mathrm{median}}\Delta p_{i}$, where $b_{c}\in R^{+}$ and $b_{p}\in R^{+}$. We kept an option if its spread was below the scaled median. Note that a complete option pair was required to compute the synthetic forward price, and, hence, if the one option in the pair was removed, the other one became useless. The parameters used to generate all out-of-sample results are presented in Table 8.

#### 4.3.2. Evaluation Method

It is critical to choose how to evaluate an estimate. One approach would be to follow the path used by Desmettre et al. [1]. They estimated the dividends for individual shares and compared the results of their estimates with the realized dividends, but we argue that this approach has some intrinsic drawbacks. First, Desmettre et al. [1] discuss a difference in their estimates of the market consensus of the present value dividends and the actual dividends. They used market data for specific markets with a tax setting that they argued was suitable. This favorable tax setting is not present in the US market, and, in Section 2.1, we argue that we do not measure the dividends but rather how the index is affected by them. Second, there is also a practical problem with index data. The index does not pay dividends but rather its constituents, which results in considerably more dividend payments, and the payments must be scaled with the weight of its constituents. All these technical details make the method error-prone and thus not suitable for use. To summarize, even in idealistic conditions, it is not generally valid to compare dividend estimates with their realized counterparts.

Another natural approach would be to use the linear models and the predicted errors, which, in essence, is how well the linear models reprice the out-of-sample options. The advantage of the prediction errors is that they are easily computable and allow an easy model comparison. The primary disadvantage is that the linear regressions of the put–call parity also include estimations of the interest rate spread. Hence, prediction errors are affected by both the quality of the dividends and the interest rate spreads estimates. The results are, thus, in a strict sense, a measure of linear model performance, but not necessarily of the dividend. Consequently, we base our estimate on another approach: utilizing the sloped asset position. The limitation of using this position as an estimator is the vast amount of combinations. A potential solution to this limitation is to limit the data, but the drawback is figuring out how to make such a limitation systematically. However, in the out-of-sample testing, the data set was, by construction, small enough to use sloped asset positions. The sloped asset position makes it possible to test the estimates isolated from the potential effects of the interest rate. Furthermore, we use the adjusted share price formulation, $S^{*}$, to compare yield and present value since the two types are not directly comparable. The regressions were run in-sample, and they were then compared with the help of the out-of-sample data.

The mean squared errors of the residuals is one method of measuring and comparing the different methods. It was difficult to argue if the difference between methods was big or small. Therefore, we complemented the mean squared error with a statistical test on the out-of-sample data. We use the version of the Diebold–Mariano test that was used by Blomvall et al. [25]. This test is a version of the original test presented by Diebold and Mariano [37]. The test consists of four steps. First, we partitioned the data into in- and out-of-sample sets. Second, the regression was performed (in-sample). Third, the linear models were evaluated on the out-of-sample data to measure the errors. Fourth, we performed the Diebold–Mariano test from the errors. We denoted the errors for the two regressions, which we compared using $s_{i,1}$ and $s_{i,2}$, respectively, where i=1,…,n. Let $d_{i}=s_{i,1}i^{2}-s_{i,2}^{2},\;\forall i=1,\ldots ,n$ denote the loss differentials, and let

$$\bar{d}=\frac{1}{n}\sum_{i=1}^{n}d_{i},$$

denote the mean of the loss differentials, and the autocovariance with lag *k* be

(31) $$\gamma_{k}=\frac{1}{n}\sum_{i=k+1}^{n}\left(d_{i}-\bar{d}\right)\left(d_{i-k}-\bar{d}\right).$$

The Diebold–Mariano statistic was formulated as

(32) $$DM=\frac{\bar{d}}{\sqrt{\frac{1}{n}\left(\gamma_{0}+2\sum_{k=1}^{h-1}\gamma_{k}\right)}},$$

where $h\in N^{+}$, i.e., a strictly positive integer, and we chose $h=n^{1/3}+1$. The Diebold–Mariano test statistic follows a standard normal, N(0,1), given the null hypothesis $H_{0}:$$E\left[d_{i}\right]=\mu =0.$ We computed the errors, $s_{i,1}$ and $s_{i,2}$, in two ways. First, we used the prediction errors of the linear models of the out-of-sample data. Second, we also used the adjusted spot price that was implied by the sloped asset position.

Further, the Diebold–Mariano test only determines if there is a (significant) difference between methods, but the test does not quantify this difference. However, Blomvall et al. [25] present one measure, $\sqrt{2/\pi }\sqrt{\bar{d}}$, that can be interpreted as the average improvement between estimates. The measure has the same unit as the errors, $s_{i,1}$ and $s_{i,2}$.

#### 4.3.3. Results

The results are divided between the mean squared errors presented in Table 9 and the Diebold–Mariano tests presented in Table 10. The Diebold–Mariano tests cover the comparisons between yield and present value, ordinary and weighted least squares formulations, and single-time and intraday data. Table 9 shows that the out-of-sample results are consistent with the in-sample results, since the present value outperformed the yield formulation. Moreover, the weighted least squares formulation performed better than the ordinary least squares formulation. The mean squared errors indicate the performance of the different models, but they do not quantify the significance or even if the difference is significant.

To see the statistical significance between the models, we discuss the Diebold–Mariano results in this section. The Diebold–Mariano test results are presented in Table 10. That table presents the test statistic, and all the comparisons show significant differences. Further, the fifth column, $\sqrt{2/\pi }\sqrt{\bar{d}}$, is a measure of the differences between the methods. The statistical test and the values of the measures yield the same results, which can be summarized in three points. First, the present value dividend formulation is significantly better than the yield formulation, and the improvements are between 13.91 to 17.61 cents. Second, the weighted least squares formulation is significantly better than the ordinary least squares formulation, and the improvements are between 3.95 to 19.37 cents. Third, basing dividend estimates on intraday data is significantly better than single time data, and the improvement is 54.57 cents. These quantitative results align with the earlier qualitative results.

### 4.4. Performance Difference between Yield and Present Value

We have seen that the present value formulation has a superior performance to the yield formulation both in-sample and out-of-sample for intraday data. If single-time data is used, there is no difference between the two formulations. The methods are similar in assumptions but with a crucial difference. The dividend quantity is assumed constant in both regressions, but a constant dividend yield implies different adjustments to the spot price, which is incompatible with the market participants’ perception.

We tested if this variability in the adjustment can explain the inferior performance. We performed a regression that related the difference between the methods and the intraday variability of the spot price to each other. It is possible to create many variability measures, but there are two features that we would like the measure to have. First, the absolute quote changes are less interesting than the relative changes, i.e., the changes should be related to spot price. Second, we want the regression to be easily computable and tractable.

It is also possible to create several measures of dividend differences. We chose to measure the intraday variability of the spot price as the intraday range of the spot price divided by the median spot price. First, we introduced times $t_{j}^{\prime },\;j=1,\ldots ,M^{d}$, which were the times when the spot price of the index was recorded, and $M^{d}$ was the number of such times for day *d*. The variability of the spot price for a day *d* was then written as

(33) $$\Delta S_{d}=\frac{\max_{j\in \{1,\ldots ,M^{d}\}}S\left(t_{j}\right)-\min_{j\in \{1,\ldots ,M^{d}\}}S\left(t_{j}\right)}{\underset{j\in \{1,\ldots ,M^{d}\}}{\mathrm{median}}S\left(t_{j}\right)},$$

and the difference between the dividends of the two as

(34) $$Y_{d}\left(T\right)=\frac{1}{T}\left|{\hat{D}}^{d,T}-D_{y}^{d,T}\right|.$$

where

(35) $${\hat{D}}_{y}^{d,T}=\underset{j\in \{1,\ldots ,M^{d}\}}{\mathrm{median}}S\left(t_{j}\right)\left(1-{\hat{\gamma }}_{2}^{d,T}\right).$$

**Figure 5:**
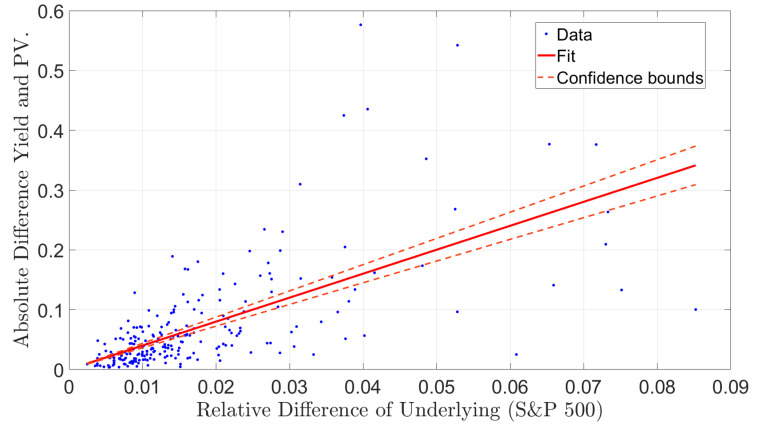
This figure illustrates the regression results between the variability of the underlying and the difference between estimating a dividend yield and a present value of a dividend. The slope coefficient is 4.0059, which means that the greater variability predicts a bigger difference between the yield and the present value estimate.

The values of ${\hat{D}}_{y}^{d,T}$ were aggregated into a single value, $Y_{d}$ for each date as the mean of $Y_{d}\left(T\right)$. The regression can then be formulated as

(36) $$Y_{d}=\beta \Delta S_{d},$$

and the results are presented in Table 11 and Figure 5. We can see that the t-statistic indicates that the coefficient is significantly different from zero, indicating that the spot price variability partly explains the difference. Furthermore, we can see from Figure 5 that the spot price variability is probably not the sole explanation, but it is possible to conclude that increased variability increases the difference between the two dividend formulations.

### 4.5. Conclusions

This paper has made both practical and theoretical contributions to the literature in this area. The practical contribution is that we have expanded and generalized the regression method presented by Desmettre et al. [1] in two regards. First, we have generalized the regression from an ordinary least squares formulation to a weighted least squares formulation. Second, the regression has been reformulated to utilize intraday data rather than being limited to data recorded at a single time. We have proven that both of these changes improve the quality of the dividend estimates with statistical significance. The latter improved the estimation more than the former. Additionally, one key component of this analysis is the new European option position (the sloped asset position) that we have introduced. This position makes it possible to evaluate dividend estimates independent of interest rate estimates.

The main theoretical contribution is that we have proven that the present value dividend formulation performs significantly better than the yield formulation. We have also proposed an explanation for this phenomenon. We propose that worse performance is caused by the inherent connection between the yield and the spot price. We have also contributed theoretically with the clarification of the interpretation of the dividend. These realizations could affect, e.g., the dividend adjustments in derivative pricing.

## Figures and Tables

**Table 1:** The two panels schematically exemplify mock tick market data of two assets. The marker N.U. indicates Not Updated.

Time	Bid Quote (1)	Ask Quote (1)	Time	Bid Quote (2)	Ask Quote (2)
09:01:02	100	102	09:02:00	200	N.U.
09:02:30	N.U.	103	09:02:59	N.U.	213
09:03:15	99	N.U.	09:04:10	199	N.U.
⋮	⋮	⋮	⋮	⋮	⋮

**Table 2:** The two panels schematically exemplify one-second data that have been derived from Table 1. The left and right panels are derived from the left and right panels in Table 1, respectively. The bold quotes indicate that those quotes were ticks and not repeats of an earlier tick. Bold times indicate that, at that time, at least one of the quotes (bid and ask) was a tick.

Time	Bid Quote (1)	Ask Quote (1)	Time	Bid Quote (2)	Ask Quote (2)
09:01:02	100	102	09:02:00	200	n/a
⋮	⋮	⋮	⋮	⋮	⋮
09:02:29	100	102	09:02:58	200	n/a
09:02:30	100	103	09:02:59	200	213
⋮	⋮	⋮	⋮	⋮	⋮
09:03:14	100	103	09:04:09	200	213
09:03:15	99	103	09:04:10	199	213
⋮	⋮	⋮	⋮	⋮	⋮

**Table 3:** This table schematically exemplifies one-second data, which combines the two panels in Table 2. The bold numbers indicate that those numbers were ticks in the tick data. (Note that every row has at least one bolded number) The difference between the panels in Table 2 and this table is that times that lack a tick have been removed from this table.

Time	Bid Quote (1)	Ask Quote (1)	Bid Quote (2)	Ask Quote (2)
09:01:02	100	102	N/A	N/A
09:02:00	100	102	200	N/A
09:02:30	100	103	200	N/A
09:02:59	100	103	200	213
09:03:15	99	103	200	213
09:04:10	99	103	199	213
⋮	⋮	⋮	⋮	⋮

**Table 4:** This table shows statistics for the daily differences in market-matched present value dividend estimates for four series of estimates with a constant time of maturity. The numbers (382, 473, 655, 1019) in the first column—TTM—are the times to maturity on the 2 March 2020 (i.e., the first date in the data set.) for the series.

	Single-Time			Intraday		
TTM	Mean	Std	Autocorr	Mean	Std	Autocorr
382	−0.24581	1.7569	−0.1491	−0.23592	1.1137	0.33601
473	−0.25063	1.9994	−0.053606	−0.23916	1.387	0.31847
655	−0.26461	3.3015	−0.17999	−0.25003	2.1288	0.28339
1019	−0.24773	5.7669	−0.32063	−0.23198	3.5832	0.12227

**Table 5:** This table consists of mean squared errors (MSE) for in-sample dividend ordinary least squares (OLS) estimates based on intraday and single-time (recorded at 3 p.m.) data. The table shows the mean squared errors for the yield and present value dividend formulation. The MSE for the single-time data is—by construction—equal for both dividend formulations; hence, these are written on the same row.

Dividend Type	Data	MSE
Yield	Intraday	3.2104
Present value	Intraday	3.1806
Yield and Present value	Single-time	3.2114

**Table 6:** This table presents the statistics of the differences between the present value estimates and the implied present value quantity, $D_{y}$. The second column—Abs. Mean—shows the values of the mean of the absolute value of the differences. The last row—All TTM—shows the statistics for all differences. The other rows show groups of differences corresponding to the times to maturity (days) in the range.

TTM Range [Days]	Abs. Mean	Mean	Std. Dev.	Skewness	Kurtosis
[0,100)	0.0314	−0.00567	0.0587	−1.6364	27.3525
[100,200)	0.0746	−0.00391	0.1244	−0.6311	16.1054
[200,300)	0.1385	−0.00397	0.2241	−0.4728	11.2769
[300,400)	0.1636	0.00575	0.2648	−0.3781	12.5705
[400,500)	0.2032	0.01219	0.3175	−0.2575	11.8386
[500,600)	0.1918	0.03577	0.2692	0.1181	6.7503
[600,700)	0.3390	0.02911	0.5131	−0.3406	9.1445
[700,800)	0.2405	0.05065	0.3331	0.2678	6.0934
[800,900)	0.2991	0.06862	0.4279	0.0755	6.7246
[900,1000)	0.4294	0.05606	0.5963	−0.0068	5.9678
[1000,1100]	0.6999	0.02285	1.0509	−0.5342	7.0079
All TTM	0.1363	0.00642	0.2727	-0.4462	37.0126

**Table 7:** This table shows the statistics for the daily differences of the stripped present value dividend estimates for four series of estimates with a constant time of maturity. The intervals (e.g., 473–382) in the first column—Tenors (TTM)—indicate time to maturity intervals on 2 March 2020 (the first date in the data set) that the stripped dividend estimates correspond to.

	Single-Time			Intraday		
Tenors (TTM)	Mean	Std	Autocorr	Mean	Std	Autocorr
473–382	−0.0048	1.3296	−0.4810	−0.0032	0.6636	−0.2873
655–473	−0.0140	2.8049	−0.4661	−0.0109	1.1899	−0.0508
1019–655	0.0169	4.2557	−0.4981	0.0181	2.0258	−0.1932

**Table 8:** This table shows the parameters used to partition the data set into in- and out-of-sample data sets.

Parameter	Value
$\ell^{\prime }$	0.75
$u^{\prime }$	1.25
*k*	10
$b_{c}$	0.30
$b_{p}$	0.30

**Table 9:** This table shows the out-of-sample mean squared errors (MSE) of the ordinary least squares (OLS) the weighted least squares (WLS) formulations and the differences between these two regressions. This table presents the results of two different measures: dividend yield and present value dividend; and two evaluation methods: regression residuals and difference to the sloped asset position.

Dividend Formulation	Data	MSE		
		OLS (MSE)	WLS (MSE)	OLS − WLS
Present Value	Intraday Prediction	0.2826	0.2800	0.0026
Yield	Intraday Prediction	0.3130	0.3105	0.0025
Present Value	Intraday Sloped	32.609	32.597	0.0118
Yield	Intraday Sloped	32.656	32.646	0.0103

**Table 10:** This table presents the Diebold–Mariano test statistics for the comparison between the different estimation methods. The first three columns show information about the method; the type of dividend formulation: yield or present value (PV); the regression form: ordinary (OLS) or weighted least squares (WLS); the type of data used: either single-time (Single) or intraday (I-day) data; and the errors that can be based on prediction or sloped asset positions. The Diebold–Mariano test compares a pair of methods, and each row in the table is one such comparison, and the compared methods are indicated with “reference method” vs. “alternative method”. For example, in the first row, the yield and present value formulation are compared. The fifth and sixth rows contain the mean of the differential and the Diebold–Mariano test statistics, where a positive or negative sign indicates that the alternative method is better or worse, respectively, than the reference method.

Dividend Formulation	Regression Formulation	Data	$\bar{d}$	$\sqrt{2/\pi \bar{d}}$ [USD]	DM
Yield vs. PV	OLS	I-day Predicition	0.0304	0.1391	22.710
Yield vs. PV	WLS	I-day Predicition	0.0305	0.1394	23.220
Yield vs. PV	OLS	I-day Sloped	0.0472	0.1733	37.096
Yield vs. PV	WLS	I-day Sloped	0.0487	0.1761	37.998
PV	OLS vs. WLS	I-day Prediction	0.0025	0.0395	4.2099
PV	OLS vs. WLS	I-day Sloped	0.0590	0.1937	23.408
PV	OLS	Single vs. I-day Sloped	0.4660	0.5447	79.258

**Table 11:** This plot is the result of regressing and understanding the problem with estimating the dividend yield.

	Estimate	SE	t-Statistic
β	4.0059	0.1920	20.8637

## Data Availability

All the data have been collected from the data provider Thomson Reuters Refinitiv Eikon.

## Appendix A

The derivation of the *sloped asset* position can be made either with a dividend expressed as a yield or as a present value of future dividends. Here, we derive both versions. Let, $f_{i}$ be a synthetic forward with strike price $K_{i}$, i.e., the options in the option pair used to construct the synthetic forward have the strike price $K_{i}$. Furthermore, let δ and *D* denote the dividend yield and present value of the dividends, respectively. Furthermore, let *r* denote the continuous interest rate. However, the rate is not necessary to prove the relation. Finally, let *T* denote the time to maturity when entering the contracts, and the time the contract is entered into is *t*.

The sloped asset position consists of a long position in $f_{1}$ and $\frac{K_{1}}{K_{2}}$ short positions in $f_{2}$. The former has a payoff that can be written as $g_{1}\left(s\right)=s-K_{1}$, while the short positions provide a payoff of $g_{2}\left(s\right)=-\frac{K_{1}}{K_{2}}\left(s-K_{2}\right)=\frac{K_{1}}{K_{2}}\left(K_{2}-s\right)$. The total payoff at expiration for the complete position is:

$$g\left(s\right)=g_{1}\left(s\right)+g_{2}\left(s\right)=\left(s-K_{1}\right)+\left(\frac{K_{1}}{K_{2}}\left(K_{2}-s\right)\right)=s\left(1-\frac{K_{1}}{K_{2}}\right).$$

The payoff can be interpreted as a fractional position, either long or short, in the shares. The price of this contract upon entering is the share price adjusted for dividends (scaled with the factor), i.e., the following:

(A1) $$\begin{array}{cc} f_{1}-\frac{K_{1}}{K_{2}}f_{2} & =S^{*}\left(1-\frac{K_{1}}{K_{2}}\right)=Se^{-\delta (T-t)}\left(1-\frac{K_{1}}{K_{2}}\right) \end{array}$$

(A2) $$\begin{array}{cc} f_{1}-\frac{K_{1}}{K_{2}}f_{2} & =S^{*}\left(1-\frac{K_{1}}{K_{2}}\right)=\left(S-D\right)\left(1-\frac{K_{1}}{K_{2}}\right). \end{array}$$

It is possible to find the dividend yields and the present values of the dividends directly from the expressions by rearranging them thus:

(A3) $$\begin{array}{cc} \delta & =-\frac{1}{T-t}\ln\left[S^{*}/S\right]=-\frac{1}{T-t}\ln\left[\frac{1}{S}\frac{f_{1}K_{2}-f_{2}K_{1}}{K_{2}-K_{1}}\right], \end{array}$$

(A4) $$\begin{array}{cc} D & =S-S^{*}=S-\frac{f_{1}K_{2}-f_{2}K_{1}}{K_{2}-K_{1}}. \end{array}$$
